# Common mental disorders among workers chronically exposed to pesticides: the case of workers involved in fighting endemic diseases

**DOI:** 10.5327/Z1679443520190455

**Published:** 2019-12-01

**Authors:** Maria Luiza Almeida Bastos, Marcos Clint Leal de Carvalho, Marcelo José Monteiro Ferreira, Thiago Holanda Freitas, Geraldo Bezerra da Silva-Junior

**Affiliations:** 1 School of Medicine, Graduate Program in Public Health, Universidade Federal do Ceará - Fortaleza (CE), Brazil. Universidade Federal do Ceará School of Medicine Graduate Program in Public Health Universidade Federal do Ceará Brazil; 2 Graduate Program in Collective Health, Universidade de Fortaleza - Fortaleza (CE), Brazil. Universidade de Fortaleza Graduate Program in Collective Health Universidade de Fortaleza Brazil

**Keywords:** mental disorders, pesticides, community health agents, public health

## Abstract

**Background:**

Common mental disorders are a considerable global public health concern and pose a high burden of disease especially in terms of years lived with disability (YLD).

**Objective:**

To establish the prevalence of common mental disorders among workers involved in fighting endemic diseases in the state of Ceará, Brazil.

**Methods:**

In the present cross-sectional study we administered the Self-Reporting Questionnaire 20, a screening tool for common mental disorders previously validated for use in Brazil. To calculate the sample size we considered the total number of workers involved in fighting endemic diseases in Fortaleza and four metropolitan municipalities.

**Results:**

About 33.3% of the participants met the criteria for common mental disorders. The questionnaire section on depressive and anxiety symptoms exhibited the highest frequency of affirmative answers. The prevalence ratio was highest for the participants in Caucaia (PR=2.13).

**Conclusion:**

The results indicate a previously unknown and considerable morbidity profile. The present was the first study that investigated common mental disorders in this occupational group.

## RESUMO |

**Introdução:**

Os transtornos mentais comuns correspondem a importantes problemas de saúde pública e conferem alta carga de doença.

**Objetivo:**

Investigar a prevalência de transtornos mentais comuns nos agentes de combate a endemias no estado do Ceará.

**Métodos:**

Foi realizado estudo de corte transversal, com instrumento de rastreio para transtornos mentais comuns validado no Brasil, o Self-Reporting Questionnaire (SRQ-20). Para o cálculo do tamanho da amostra, foi considerado o número total de trabalhadores ocupantes do cargo de agente de combate a endemias lotados em Fortaleza e em quatro municípios da região metropolitana.

**Resultados:**

Foi identificado que 33,3% dos trabalhadores apresentaram critérios para transtornos mentais comuns. As respostas mais prevalentes corresponderam ao bloco de perguntas dos componentes humor ansioso e depressivo. Os trabalhadores que labutam no município de Caucaia apresentaram razão de prevalência superior para desenvolver transtornos mentais comuns (razão de prevalência - RP=2,13) quando comparados aos agentes de combate a endemias lotados nos outros municípios.

**Conclusão:**

Os resultados desta pesquisa evidenciaram um perfil de morbidade importante e até então desconhecido. Este estudo é pioneiro na investigação de transtornos mentais comuns no tocante aos trabalhadores da saúde pública expostos a agrotóxicos utilizados nas campanhas de combate a vetores.

## INTRODUCTION

Mental disorders are a significant global public health concern, with a lifetime prevalence of 18.1% to 36.1% among 28 representative countries considered in WHO World Mental Health surveys^1^. Mental disorders account for up to 13% of the total global burden of disease in terms of disability adjusted life years (DALY), thus similar to cardiovascular and circulatory diseases, as well as for 32.4% of the total number of years lived with disability (YLD) in which case they rank first^2^. In Brazil, mental disorders were the leading cause of disability in 2015, accounted for 24.9% of total YLD and were the third most frequent cause of DALY, 9.5% of the total^3^.

The relevance of work for mental health is widely acknowledged as a both health or illness promoting factor. On these grounds, the Brazilian System of Information on Notifiable Diseases (SINAN) included in 2004 work-related mental disorders in the list of conditions which must be mandatorily reported.

Mental disorders are the third leading reason for disability benefits granted by the Brazilian National Institute Social Security Institute and the main cause of sick leave among civil servants^4^^,^^5^.

Common mental disorders (CMD) - i.e. depressive, anxiety and somatoform symptoms, to the exclusion of psychotic conditions - are among the most frequent WRMD, with a lifetime prevalence of 29.2% as estimated in a meta-analysis of data relative to 63 countries^6^. In addition to disability, CMD are also associated with absenteeism and presenteeism^7^. In population-based studies^8^, the prevalence of CMD in Brazil varied from 17% to 35% and were detected among several occupational categories, such as mid- and high-level health care providers^9^, community health agents^9^^,^^10^, social educators^11^ and bus drivers and conductors^8^.

Workers involved in fighting endemic diseases are charged of the public control of vectors and thus are exposed to pesticides. These compounds were associated with occurrence of mental disorders in studies performed in rural communities^12^^,^^13^. CMD were reported among similar occupational categories, such as community health agents, primary care providers and social educators^9^^,^^10^^,^^11^. We considered the case of rural workers to be pertinent, because also they are occupationally exposed to pesticides^14^. Indeed, acute pesticide poisoning is one of the main risk factors for mental and neuropsychiatric disorders among the rural population^13^^,^^14^^,^^15^.

Given the failure of socioenvironmental measures to eradicate vectors and the association between occupational exposure to pesticides and neurobehavioral disorders, we believe that a study of CMD among workers involved in fighting endemic diseases in Ceará, Brazil, is relevant.

## METHODS

### STUDY DESIGN AND POPULATION

The present cross-sectional and analytical study was conducted from September 2017 to May 2018 with workers involved in fighting endemic diseases in Ceará. For this purpose we visited technical districts, i.e. the places where these workers begin their working day, from November 2017 through March 2018.

### STUDY POPULATION AND SAMPLE

For sample size calculation we considered the total number of workers hired to fight endemic diseases in Fortaleza and other four metropolitan municipalities, Cascavel, Caucaia, Horizonte and Pacajus, to a total of 374. Calculation was performed with software Epi Info, 95% confidence interval and incidence proportion of 50% since there are no data in the literature on the prevalence of CMD among this population of workers.

Eligible subjects were those in activity in the fight of endemic diseases. Workers who returned incomplete/non-responded questionnaires and those who refused participation were excluded.

### INSTRUMENTS AND VARIABLES

We administered the Self-Reporting Questionnaire 20 (SRQ-20) which has been validated for and widely used in screening for non-psychotic CMD^17^^,^^18^^,^^19^^,^^20^^,^^21^. SRQ-20 comprises 20 questions corresponding to four groups of symptoms: depressed-anxious mood, somatic symptoms, decreased vital energy and depressive thoughts. Individual question scores are summed, the total ranging from 0 to 20. Cut-off points are variably established as a function of the epidemiological characteristics of the target population^18^^,^^21^; in the present study we set score 5 as cut-off point.

### STATISTICAL ANALYSIS

The data were tabulated using software Microsoft Excel for Mac version 16.11.1. We first subject all the considered analysis to descriptive analysis. Bivariate analysis was performed with software Stata for Windows version SE 10.

### ETHICAL ISSUES

The study was approved by the research ethics committee of University of Fortaleza (Unifor) ruling no. 2,216,806/2017, and is part of a master’s research project in collective health.

## RESULTS

A total of 105 workers returned properly responded questionnaires, which represented 55.3% of the estimated sample size (n=190). Most participants were male (n=101, 96.2%), with mean age 59 years old (standard deviation - SD=4.96) and 34 years (SD=3.6) in the job, on average

The prevalence of CMD in the analyzed sample was 33.3%, being higher for the items related to depressive-anxious mood. Question #6 - “Do you feel nervous, tense or worried?” - received the highest frequency of affirmative answers, 50.5%, followed by #3 - “Do you have sleep disturbances?” - n=40, 38.1%, and #4 - “Are you easily frightened? - n=32, 30.5%.

While the largest number of workers who met the criteria for CMD were located in Fortaleza, n=23, 32%, this condition was found for half of those in Caucaia (n=4). The latter also exhibited a higher prevalence ratio for CMD (PR=2.13) but that was not statistically significant. The results of descriptive analysis according to sex and town are shown in Table 1.

**Table 1.** Sociodemographic distribution according to age and town of workers involved in fighting endemic diseases who responded the Self-Reporting Questionnaire 20, Ceará, 2017-2018 (n=105)

	CMD no			CMD yes			% workers with CMD	PR	95%CI	p value
	Male	Female	Total	Male	Female	Total				
Cascavel	7	1	8	3	0	3	38	1.22	0.27-5.42	0.76
Caucaia	8	0	8	4	0	4	50	2.13	0.5-9.08	
Fortaleza	69	3	72	22	1	23	32	0.84	0.35-1.99	
Horizonte	4	0	4	1	0	1	25	0.66	0.07-6.56	
Pacajus	13	0	13	4	0	4	31	0.87	0.25-3.07	
TOTAL	101	4	105	34	1	35	33.3%			

CMD: common mental disorders; PR: prevalence ratio; 95%CI: 95% confidence interval.

## DISCUSSION

The present is the first study that analyzed CMD among workers involved in fighting endemic diseases in Brazil. Indeed, we were not able to locate any study targeting this population of workers in the national or international literature. These workers are charged of combatting vectors, with current emphasis on arbovirus epidemics in Brazil in general, and in Ceará in particular^16^.

We found a high prevalence of CMD in the analyzed population (33.3%), superior to that of previous studies conducted with similar populations^9^^,^^22^^,^^23^. The SRQ-20 question with the highest frequency of affirmative responses was that related to depressive-anxious aspects. According to SRQ-20 validation studies, question #6 (“Do you feel nervous, tense or worried?”), related to depressive-anxious mood, is the one with the highest internal consistency, sensitivity of 89.81% and specificity of 56.71%^20^^,^^21^.

Few studies analyzed mental health among primary care workers, but the available evidence points to a high prevalence of depressive symptoms in this group^24^. We were not able to locate any study that assessed the mental health of workers involved in fighting endemic diseases. Our results indicate that the prevalence of non-psychotic CMD in this group was higher than that of other categories of primary care workers^9^^,^^23^^,35^.

We believe that some aspects of fighting endemic diseases might be related to the occurrence of depressive symptoms, the main one being exposure to pesticides, as reported in previous studies performed with other exposed occupational groups^14^^,^^26^^,^^27^. One further factor to consider is perhaps the low autonomy of workers in regard to their work, since vector control campaigns tend to occur at definite times of the year, rainy seasons in particular in the Brazilian Northeast region^16^^,^^28^^,^^29^.

The present study has several limitations. The participants refused to respond a sociodemographic questionnaire at this and later periods of field research. Then, the sample size was 55.3% of the calculated. These shortcoming notwithstanding, the results evidence considerable morbidity and point to a pressing need to implement mental health care actions targeting workers involved in the control of endemic diseases in Ceará.

## CONCLUSION

The present study was the first to investigate CMD among the target population, namely, primary care workers exposed to pesticides and possibly exhibiting signs of chronic pesticide intoxication. Additional studies are needed with greater power of inference and investigation of the causal relationship between mental symptoms and chronic pesticide intoxication.

The results of the present study corroborate the increasing trend in morbidity by mental disorders and point to the need for health promotion actions in the workplace targeting the analyzed occupational group.
